# Impact of particle size, oxidation state and capping agent of different cerium dioxide nanoparticles on the phosphate-induced transformations at different pH and concentration

**DOI:** 10.1371/journal.pone.0217483

**Published:** 2019-06-07

**Authors:** Isabella Römer, Sophie Marie Briffa, Yadira Arroyo Rojas Dasilva, Dimitri Hapiuk, Vanessa Trouillet, Richard E. Palmer, Eugenia Valsami-Jones

**Affiliations:** 1 School of Geography, Earth and Environmental Sciences, University of Birmingham, Edgbaston, Birmingham, United Kingdom; 2 Empa, Swiss Federal Laboratories for Materials Science and Technology, Electron Microscopy Center, Dübendorf, Switzerland; 3 Nanoscale Physics Research Laboratory, School of Physics and Astronomy, University of Birmingham, Edgbaston, Birmingham, United Kingdom; 4 Institute for Applied Materials (IAM) and Karlsruhe Nano Micro Facility (KNMF), Karlsruhe Institute of Technology (KIT), Eggenstein-Leopoldshafen, Germany; 5 College of Engineering, Swansea University, Bay Campus, Fabian Way, Swansea, United Kingdom; VIT University, INDIA

## Abstract

The potential hazard posed by nanomaterials can be significantly influenced by transformations which these materials undergo during their lifecycle, from manufacturing through to disposal. The transformations may depend on the nanomaterials’ own physicochemical properties as well as the environment they are exposed to. This study focuses on the mechanisms of transformation of cerium oxide nanoparticles (CeO_2_ NPs) in laboratory experiments which simulate potential scenarios in which the NPs are exposed to phosphate-bearing media. We have experimented with the transformation of four different kinds of CeO_2_ NPs, in order to investigate the effects of nanoparticle size, capping agent (three were uncapped and one was PVP capped) and oxidation state (two consisted mostly of Ce^4+^ and two were a mix of Ce^3+/^Ce^4+^). They were exposed to a reaction solution containing KH_2_PO_4_, citric acid and ascorbic acid at pH values of 2.3, 5.5 and 12.3, and concentrations of 1mM and 5mM. The transformations were followed by UV-vis, zeta potential and XRD measurements, which were taken after 7 and 21 days, and by transmission electron microscopy after 21 days. X-ray photoelectron spectroscopy was measured at 5mM concentration after 21 days for some samples. Results show that for pH 5 and 5mM phosphate concentration, CePO_4_ NPs were formed. Nanoparticles that were mostly Ce^4+^ did not dissolve at 1mM reagent concentration, and did not produce CePO_4_ NPs. When PVP was present as a capping agent it proved to be an extra reducing agent, and CePO_4_ was found under all conditions used. This is the first paper where the transformation of CeO_2_ NPs in the presence of phosphate has been studied for particles with different size, shapes and capping agents, in a range of different conditions and using many different characterisation methods.

## Introduction

Nanoparticles (NPs) can be defined as materials with at least one dimension between 1 and 100 nm, and that possess unique physicochemical properties that differ from the bulk [1–3]. The global market for nanomaterials (NMs) already exceeds 10 million tons with products underpinned by nanotechnology having a global value of €2 trillion [4]. With employment in the NMs sector at about 400,000 in Europe alone, the industry contributes significantly to the economy and its products are improving the quality of human life [4]. Due to the expanding use of NMs in products their discharge to the environment is rapidly increasing and having knowledge of how they behave and change under different conditions is very important [5,6]. Most published work to date has focused on pristine NPs, which can be structurally and chemically distinct from their aged counterparts and may behave differently and have different toxicity [7].

Cerium oxide nanoparticles (CeO_2_ NPs) have a broad range of industrial applications, including as additives in glass and ceramics, fuel-cell materials, in the automotive industry as a catalyst in diesel, as a polishing material or as a UV blocking agent [8–11]. Due to these widespread applications, CeO_2_ NPs are likely to come into contact with natural sinks such as soil and sediments. For this reason, CeO_2_ NPs have been used for a number of plant studies [11–13].

A feature of CeO_2_ NPs that influences their likely lifecycle is their chemical stability; they are generally considered to be sparingly soluble in aqueous media, before and after uptake by animals or plants [2,14]. It is generally accepted that in its bulk form CeO_2_ has a fluorite structure found in 99.99% of the material, which is a very stable configuration, and this accounts for its limited solubility [15]. CeO_2_ NPs have a larger specific surface area than the bulk and have the ability to cycle between oxidation states (Ce^3+^ and Ce^4+^) with very little required energy [2,16], and this depends on the oxygen partial pressure and the pH in the surrounding medium [17]. It has been observed that Ce^3+^ oxides can be soluble, contrary to what has been observed for Ce^4+^ oxides [18,19].

Most NPs used in commercial applications are mass produced, uncapped, have large size distributions [20,21], and are likely to aggregate or dissolve when exposed to natural systems releasing ions, NPs and small aggregates, all of which are likely to stay in the environment, and large aggregates, which will sediment in water [2]. The presence of a surface coating on manufactured NPs may significantly modify their surface chemistry, compared with the uncoated equivalents [22]. Coating NPs with biocompatible/organic polymers increases dispersion/stability, decreases nonspecific interactions with cells and proteins and reduces their toxicity [23–25].

Different chemicals can react with CeO_2_ NPs following environmental release or interactions with organisms, inducing dissolution or chemical transformations; phosphate in particular can be found in nutrient solutions and soils, and could lead to formation of cerium phosphate (CePO_4_) NPs, which have a high chemical stability and low expected toxicity [26,27]. A number of reports have described how the interaction of phosphate anions with CeO_2_ NPs diminishes the superoxide dismutase-mimetic activity while increasing the observed catalase-mimetic activity [28–30]. Zhang et al. (2012) found that organic acids, such as citric acid, promoted CeO_2_ NPs dissolution, and that reducing substances (ascorbic acid) played a key role in the transformation process, generating Ce^3+^ ions which then reacted with phosphate in the media [14]. It has been shown that phosphates/phosphorous bind preferentially to CeO_2_ NPs with excess Ce^3+^ sites in comparison to CeO_2_ NPs with excess Ce^4+^ sites [31]. Dahle et al. (2015) studied the dissolution of CeO_2_ NPs and found this was only significant at pH < 5, while the dissolution rate was inversely proportional to the surface area of the NPs studied [19]. Our aim in the present work was to assess if factors beyond the pH, notably concentration of the phosphate, the size of the NPs, different oxidation states and different capping agents play a role in the formation of CePO_4_ NPs.

We selected four different types of CeO_2_ NPs: Ce NM-211 and Ce NM-212 from the Joint Research Center (JRC) repository [32], uncoated CeO_2_ NPs from a commercial source [33], and PVP-capped CeO_2_ NPs synthesised in the Birmingham lab [2,34]. We used two different phosphate solution concentrations (1 and 5 mM) and three different pH values: 2.3, 5.5 and 12.3. The concentrations of phosphate were chosen to assess the effect of moderate versus high phosphate concentration; 1mM phosphate is used in Hoagland hydroponic solution [35] and 5mM would have an excess of phosphate. Cells and tissues are likely to contain high amounts of phosphate which could have a substantial influence on the biological activity of CeO_2_ NPs [30]. Organic matter, or an artificial reducing agent, must be added to a hydroponic solution to create a strong oxygen demand similar to that of flooded soils [36], which is why we added citric and ascorbic acid. Hydroponic systems and non-aerated soils, such as wetlands, are generally present in a reducing environment [37], while plant roots also secrete reducing substances, such as catechol and reducing sugars [14]. The pH values used were chosen to promote different phosphorus speciation, in the root zone this element can be found as PO_4_^3-^, HPO_4_^2-^, and H_2_PO_4_^-^ ions; the last two ions are the main forms of phosphorus taken up by plants [36]. The dependence of the speciation of phosphorus on pH is shown in S1 Fig, supporting information (SI), where it can be observed that at pH 2 H_3_PO_4_ and H_2_PO_4_^-^ are found, while at pH 5 100% of phosphorus is present as H_2_PO_4_^-^; and at pH 12 HPO_4_^2-^ and PO_3_^4-^ are found. The largest amount of phosphate available in a nutrient solution is presented when its pH is slightly acidic (pH 5) [36]. The transformations of the different CeO_2_ NPs when subjected to these various conditions of phosphate concentration and pH were followed by UV-vis, zeta potential and X-ray diffraction (XRD), which were measured after 7 and 21 days of static incubation at room temperature and in the dark, and by transmission electron microscopy (TEM) after 21 days; X-ray photoelectron spectroscopy (XPS) was measured at 5mM concentration after 21 days.

## Methodology

### CeO_2_ nanoparticles

Four different CeO_2_ NPs were used and their main properties and characterization data are shown in Table 1. Two CeO_2_ NPs were provided as a powder from JRC nanomaterial repository (Ispra, Italy) and as a part of a FP7 funded project (NanoMILE, www.http://nanomile.eu-vri.eu/) with the code name Ce NM-211 and Ce NM-212 [32]. Particles were dispersed in ultrahigh purity (UHP) water to have a final concentration of 10 mg/ml to create a stock suspension. The protocol was adapted from the nanogenotox protocol [38] as follows: the sample was weighed, UHP water was added and it was vortexed for 2-3mins. A probe sonicator (Sonics & materials INC; model vcx130; ultrasonic processors– 130 W; resonance frequency of probe– 20 kHz) was then used with an amplitude of 75% and cycle time of 0.5, Ce NM-211 was sonicated for 1 min and Ce NM-212 for 5 mins, which was found to be the minimum treatment time beyond which no further reduction in mean particle size was observed by dynamic light scattering (DLS).

**Table 1:** Test substances used, characterization performed in the lab.

Code	Test material	Surface chemistry	Core size- nm (STEM)	Hydrodynamic diameter (DLS)–nm and PDI	Zeta potential at pH 7 (mV)
Ce NM-211	Cerium (IV) oxide	Uncoated, produced by precipitation, yellowish powder, spherical	5.4 ± 2	346 ± 14 (0.68 ± 0.05)	43 ± 1
Ce NM-212	Cerium (IV) oxide	Uncoated, produced by precipitation, yellowish powder, cubic	17 ± 10	258 ± 14 (0.51 ± 0.08)	56 ± 1
PROM-Ce	Cerium (III, IV) oxide	Uncoated, produced by hydrothermal synthesis, yellowish solution, 3.1%, spherical	4.7 ± 1	172 ± 2 (0.272 ± 0.009)	50.3 ± 0.7
Ce10	Cerium (III, IV) oxide	10K PVP-capped, prepared by hydrothermal method, yellow solution, 2mg/L, spherical	7 ± 2	4.2 ± 0.2 (0.21 ± 0.02)	0.43 ± 0.03

We also used uncapped CeO_2_ NPs (PROM-Ce) as a 3.1% suspension obtained from a commercial source [33], supplied as part of the NanoMILE project and produced by a continuous one-step hydrothermal synthesis [39]. Finally, a 4^th^ set of particles, PVP-capped CeO_2_ NPs (Ce10) were produced in house, by using a published methodology for the synthesis of PVP-capped CeO_2_ NPs, using 10K PVP as the capping agent [2,34].

### Ageing of CeO_2_ NPs with phosphate at different pH

The particles were added to two different solutions of 1 mM and 5 mM of KH_2_PO_4_, citric acid and ascorbic acid, similar to the conditions used by Zhang et al. (2012), and the pH was adjusted to 2.3, 5.5 and 12.3, similar to those used by Dahle et al. (2015).

The final concentrations of the Ce NM-211, Ce NM-212 and PROM-Ce in the suspensions were 496 mg/L, used for TEM measurements, and 6200 mg/L, used for XRD measurements; and 0.4 mg/L for the Ce10 (due to having a low initial concentration). Two concentrations were used due to the fact that at least 200mg of powder was needed for XRD. After 7 and 21 days of static incubation, the highest concentration suspensions were dried out in an oven at 50°C for three days and used to measure XRD. For Ce10, we did not obtain any XRD peaks due to the combined fact of the small size of the particles coupled with the excess PVP masking any peaks. The less concentrated suspensions were used for UV-vis and zeta potential, at 7 and 21 days, and for TEM observation, after 21 days.

Time points were selected based on papers where plants were exposed to particles, Zhang et al. (2012) exposed cucumber plants to CeO_2_ nanoparticles for 21 days [14], in a different study Wang et al. exposed lettuce to CeO_2_ NPs for 10 days [40]. We observed that before 7 days of exposure no significant changes were found (in preliminary studies not shown).

### Characterisation

XRD analysis was performed using a Powder Diffractometer Bruker D8 Autosampler, with a current voltage of 40 kV, 30 mA, an X-ray source of Cu Kα, 1.5406 Å, a slit size of 1 mm, and a transmission measurement geometry. Reactions of three of the four NPs, namely Ce NM-211, Ce NM-212 and PROM-Ce, were recorded by XRD diffractograms after 21 days for the particles in 1 mM solutions and after 7 days and 21 days for the particles in 5 mM solutions.

TEM samples were prepared by partially, but not fully, drying a drop of the particle solution on a copper mesh 400 holey carbon film (Agar scientific) at room temperature [41]. The grid was washed several times with UHP water and re-dried. Images were obtained using a JEOL 1200EX (accelerating voltage 80 kV), and recorded using Gatan Digital Micrograph software. Energy dispersive X-ray spectra (EDX) were measured with a JEOL 2200FS TEM/STEM operated at 200 kV. Data was analysed using Gatan Digital Micrograph and Image J. Aberration-corrected STEM was performed on the pristine particles, as well as EELS for PROM-Ce. Images were recorded with a high angle annular dark field (HAADF) detector in a JEOL JEM2100F STEM equipped with a CEOS spherical-aberration probe corrector and a Gatan Enfina EELS.

UV-Vis absorption spectra were measured with a 6800 Jenway double beam UV-Vis spectrophotometer, collected over a wavelength range of 200–800 nm, with a 10cm long pathway quartz cuvette.

DLS and zeta potential measurements were obtained using in a Malvern Zetasizer Nano ZS. Size measurements for the pristine NPs were performed at 21°C in low volume disposable cuvettes and at least five concordant measurements were recorded to calculate a mean z-average size. Zeta potential measurements were performed at 21°C and repeated at least 5 times per sample using a low volume zeta cell which was washed with ultra-high purity (UHP) water in between each sample.

XPS characterisation was carried out at Karlsruhe Institute for Technology (KIT) in Germany. Samples were prepared by placing a drop of the 5mM PROM-Ce and Ce10 dispersions on the surface of silicon wafer, which was allowed to air-dry overnight. XPS measurements were performed using a K-Alpha+ XPS spectrometer (ThermoFisher Scientific, East Grinstead, UK). Data acquisition and processing using the Thermo Avantage software is described elsewhere [42]. All prepared samples were then analysed using a microfocused, monochromated Al Kα X-ray source (400 μm spot size). The K-Alpha+ charge compensation system was employed during analysis, using electrons of 8 eV energy, and low-energy argon ions to prevent any localized charge build-up. The spectra were fitted with one or more Voigt profiles (BE uncertainty: +0.2eV) and Scofield sensitivity factors were applied for quantification [43]. All spectra were referenced to the C 1s peak (C-C, C-H) at 285.0 eV binding energy controlled by means of the well-known photoelectron peaks of metallic Cu, Ag, and Au, respectively.

## Results and discussion

### XRD analysis

**Fig 1:**
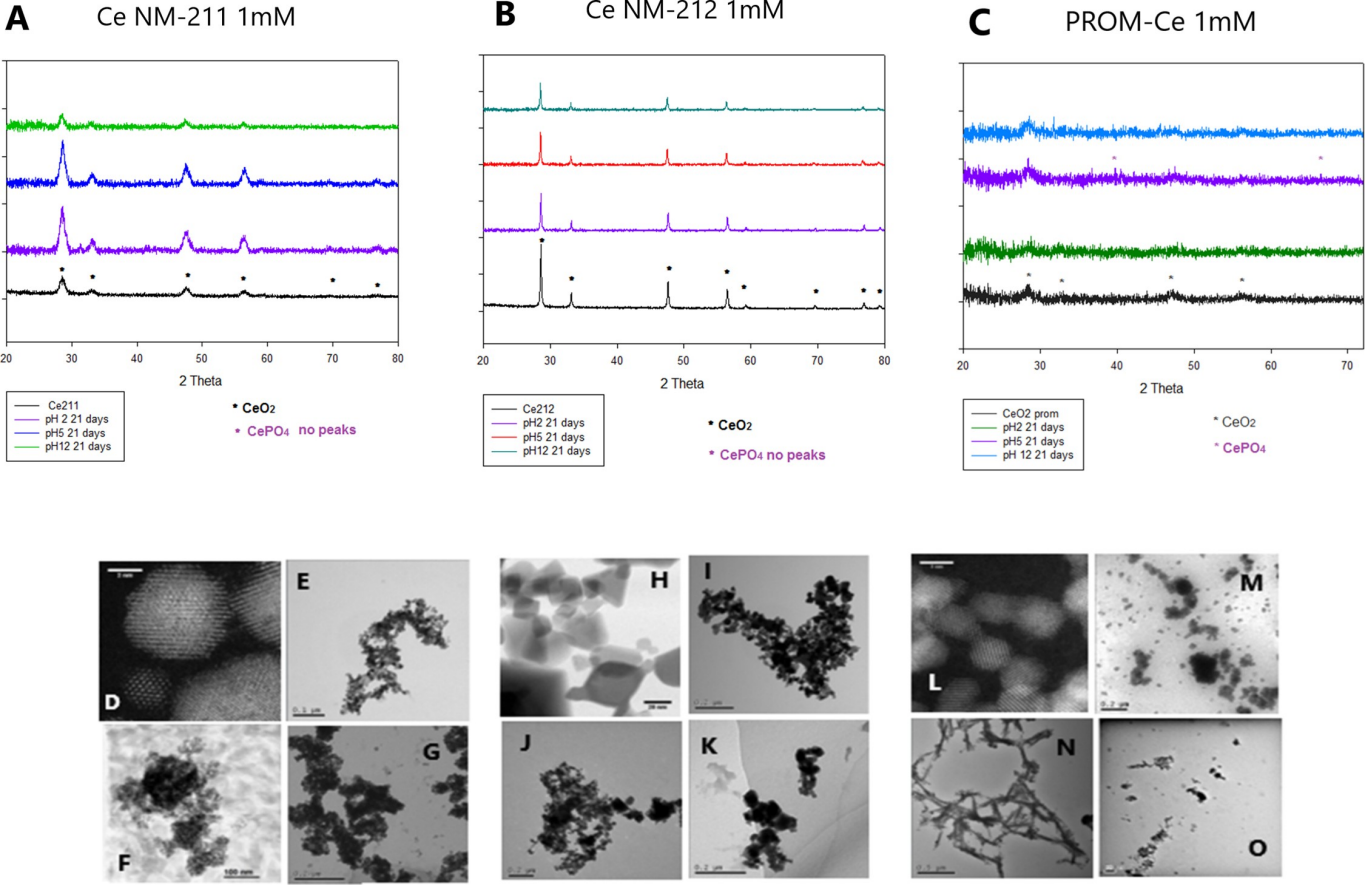
XRD and TEM results for 1mM phosphate. XRD results (top) obtained for Ce NM-211 (A), Ce NM-212 (B) and PROM-Ce (C) after 21 days at different pH in 1mM phosphate solution. TEM results obtained for Ce NM-211 (D-G), Ce NM-212 (H-K) and PROM-Ce (L-O), showing pristine NPs (D, H, L), and after 21 days at pH 2 (E, I, M), pH 5 (F, J, N) and pH 12 (G, K, O).

**Fig 2:**
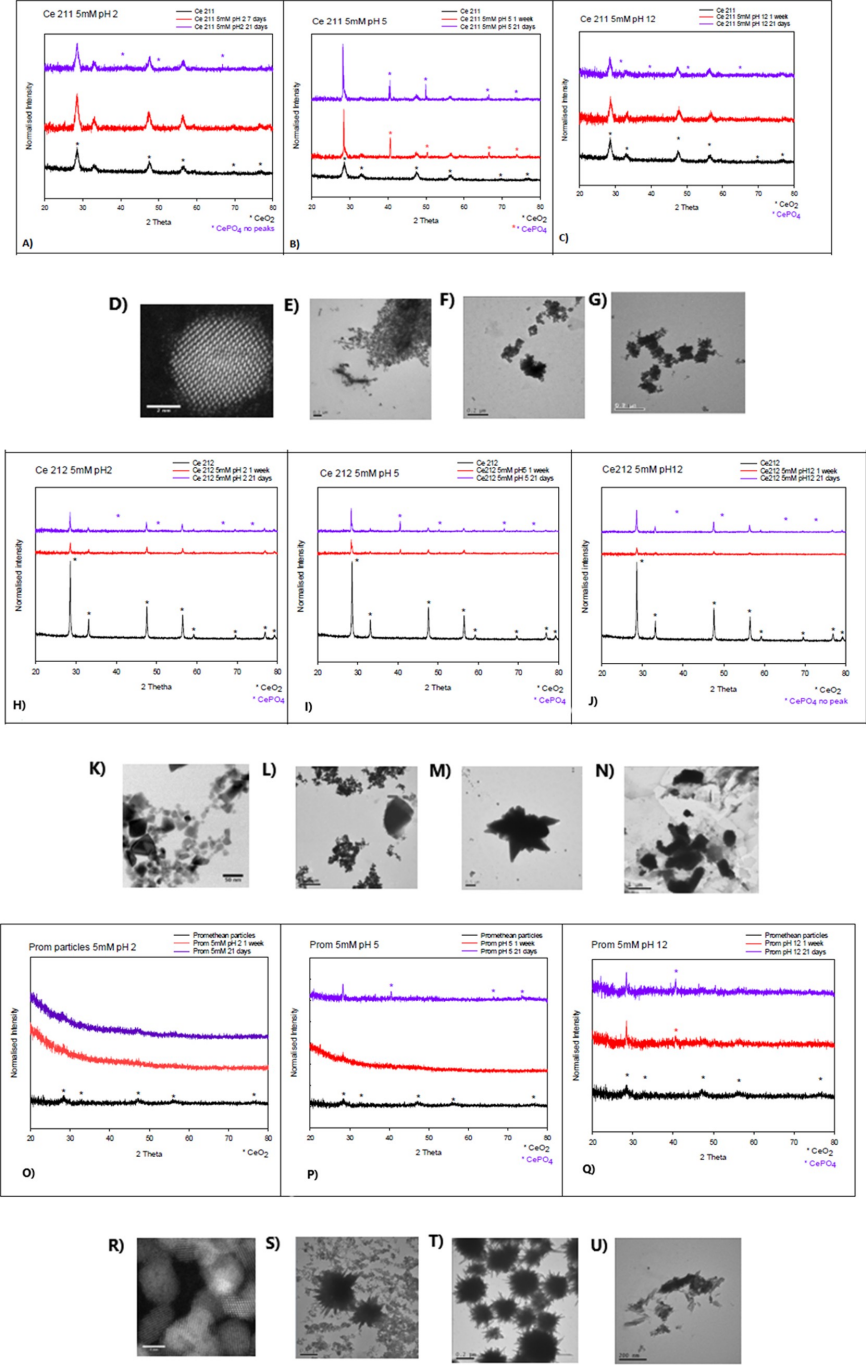
XRD and TEM results obtained for 5mM phosphate. XRD results obtained for Ce NM-211 (A-C), Ce NM-212 (H-J) and PROM-Ce (O-Q) after 1 week and 21 days at different pH in 5mM phosphate solution. TEM results after 21 days for Ce NM-211 (D-G), Ce NM-212 (K-N) and PROM-Ce (R-U), corresponding to pristine particles (D, K, R), pH 2 (E, L, S), pH 5 (F, M, T) and pH 12 (G, N, U).

Figs 1 and 2 show the diffractograms for the CeO_2_ NPs before and after ageing. Ce10 NPs were previously tested and found to produce no diffraction peaks due to the fact that the particles were too small and their weak diffraction signal was masked by the presence of PVP. For the pristine NPs, the peaks found at 2θ = 28, 33, 47 and 56° represent miller indices (111), (200), (220) and (311), respectively [44]. In the case of Ce NM-212 it can be observed that the peaks were appreciably sharper which indicates higher crystallinity [45]. Fig 1 shows the results for the particles after 21 days at different pH and 1 mM phosphate. In most cases no CePO_4_ peaks were observed, which could mean that at 1 mM concentration the reaction was not complete in most cases, except for Fig 1C at pH 5, which could be related to the initial concentration of citric and ascorbic acid.

However, at a 5 mM concentration, as shown in Fig 2, CePO_4_ peaks were observed for the particles at pH 5 after 21 days of exposure (Fig 2B, 2I and 2P), and were not observed for the particles at pH 2 (Fig 2A, 2H and 2O) and 12 (Fig 2C, 2J and 2Q), which could mean that no CePO_4_ was formed at those pH values. The CePO_4_ that seems to be formed after the exposures at 5 mM and pH 5 for Ce NM-211 and Ce NM-212 presented clear peaks at 2θ = 28, 42, 48.5 and 52° (Fig 2B and 2I), which represent miller indices (120), (-103), (103) and (-232), respectively [46]. In the case of PROM-Ce two clear peaks were observed after ageing at 5 mM and pH 5 (Fig 2P), 2θ = 28, 42°, corresponding to (120) and (-103), respectively. For PROM-Ce 1mM pH 5 (Fig 1C) only 1 peak was observed at 2θ = 42°, which corresponds to (-103) plane of CePO_4_.

These XRD results show that both concentrations of the reagents, the presence of acid and a reducing agent, and pH have an influence on the formation of CePO_4_ NPs. It is important to state that XRD is a bulk technique and results might differ from other methods used, and due to this fact more techniques are needed to assess the transformations. Another limitation of this technique is that transformations resulting in nanocrystalline or amorphous material may not produce any diffraction peaks [47].

Other studies have observed the influence of pH in the formation of CePO_4_, Li et al. (2014) observed a pH dependent biological transformation process that resulted in phosphate deposition on the particle surface of rare earth oxides (REO) and stripping of phosphate groups from the lysosomal membrane lipids [48]. In that study, they observed that CeO_2_ NPs remained substantially non-transformed, unlike other REOs studied, due to the fact that CeO_2_ was highly insoluble at both pH 7 and 4.5. Mirshafiee et al (2018) observed extremely low solubility of CeO_2_ NPs in acidic fluid as a result of its high thermodynamic stability [49]. Dahle et al. (2015) found that CeO_2_ NPs were insoluble at pH > 7 and that the addition of phosphate to CeO_2_ NPs inhibited the release of Ce species from the NPs [19]. We believe that at pH 2 and 12 the dissolution of the CeO_2_ NPs is very limited, this way there is not enough Ce^3+^ present to react with the phosphate in solution. Also, at pH 2 H_3_PO_4_ and H_2_PO_4_^-^ are found in solution, while at pH 12 we find HPO_4_^2-^ and PO_3_^4-^, which are not the most reactive phosphate speciation forms [36].

### TEM and EDX analysis

**Fig 3:**
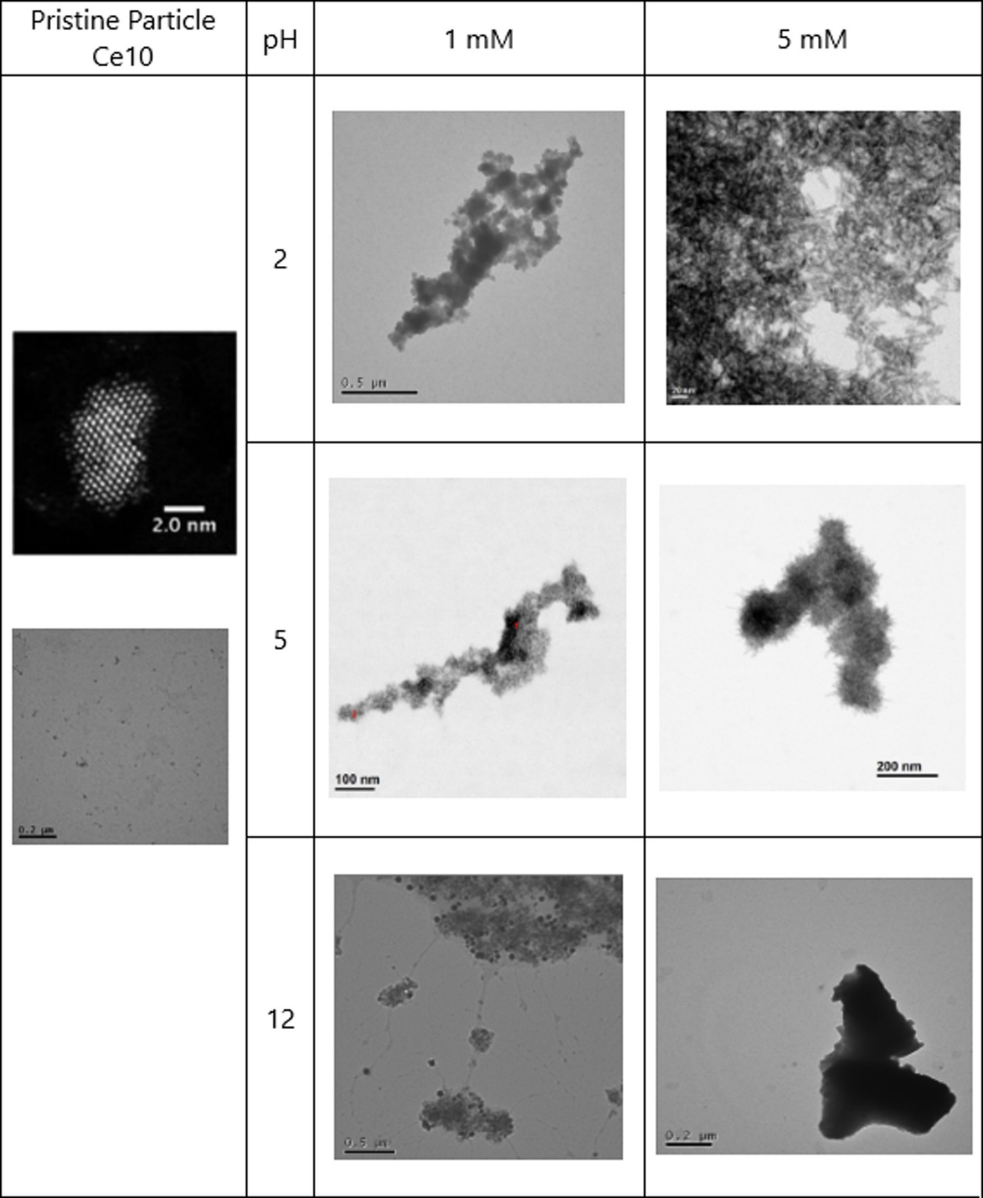
STEM and TEM images obtained for Ce10. Images obtained for the pristine Ce10 particles (STEM and TEM) and at different concentrations of phosphate and different pH after 21 days (TEM).

The particles were analysed by TEM after 21 days (Figs 1–3 and S2–S4), and samples at 1 mM pH 5 were measured with EDX (S5–S13 Figs), as well as Ce10 at 5 mM and all pH values and PROM-Ce at 5mM and pH 5. We observed aggregation of the particles in all cases, at all concentrations and pH values, as well as some physical transformations under certain conditions. Agglomeration/aggregation could have had an effect on particle dissolution and formation of CePO_4_, but this was ruled out by using two different concentrations (which were needed for the different methods) and obtaining similar results.

According to the EDX results, for Ce NM-211 and Ce NM-212 at 1 mM no phosphorus peak was observed by EDX at pH 5 (S5 and S6 Figs), which agrees with the results obtained by XRD. A phosphorus peak was observed at 1 mM and pH 5 for Ce10 and PROM-Ce (S7 and S8 Figs), and for PROM-Ce needle-like structures could be observed (Fig 1N), similar to the ones obtained by Zhang et al. (2012) under very similar conditions.

At 5 mM reagent concentration, a phosphate peak was observed by EDX for Ce10 at every pH (S9–S11 Figs), which leads us to believe that PVP could be acting as an extra reducing agent and inducing a faster and more complete dissolution of the NPs [50]. We also observed a physical transformation for Ce10 and the formation of “sea-urchin” like structures at pH5 (Fig 3), which were similar to the ones observed by Li et al. (2014) for other REOs. A phosphorus peak was also observed when PROM-Ce at 5 mM pH5 was measured by EDX (S12 Fig) as well as an even distribution of the P throughout the “sea urchin” structures observed (Fig 2T) when a compositional map, using EDX, was produced (S13 Fig). It is important to note that pH 5, which is not uncommon in acidic soils, appears to be the optimum pH to produce CePO_4_ NPs at both reagents concentrations, and leads to physical and chemical transformations for the smaller particles. In the case of larger particles where Ce^4+^ is the predominant oxidation state, Ce NM-211 and Ce NM-212, EDX showed the presence of P at 5mM and pH 5 (data not shown), which agrees with the XRD results obtained, which show a clear, albeit limited, formation of CePO_4_.

There is a significant composition difference between Ce NM-211 and Ce NM-212, compared to PROM-Ce and Ce10, which may explain why the former did not show any evidence of transformation to CePO_4_ at 1 mM pH 5 and only limited evidence at 5 mM. Whilst Ce^4+^ is the predominant oxidation state found in the JRC particles (>90%) according to the JRC repository characterization data [32], PROM-Ce is represented by a mix of Ce^3+^ and Ce^4+^ (S14 Fig), as is Ce10 [34]. It is known that Ce^4+^ oxide is less soluble than Ce^3+^ oxide [19], in the latter case, the dissolution of CeO_2_ NPs was induced by the presence of organic acids (ascorbic acid in this work), which can be secreted by plants’ roots [51]. Therefore, Ce^3+^ containing particles dissolve more easily and release cerium in the oxidation state required to react with phosphate and form CePO_4_. After dissolution, for any Ce^4+^ released, a valence change to Ce^3+^ is needed to form CePO_4_, this is a critical step according to Zhang et al. (2012) and can be achieved with the addition of reducing agents.

Nanoparticle size could have played a role in the case of Ce NM-212; Schwabe et al. (2015) found that the capacity of the CeO_2_ NPs to adsorb phosphate decreased with increasing particle size in three NP groups, in accordance with the decrease in specific surface area available for sorption [52]. Gui et al. (2015) also observed that CeO_2_ NPs with smaller size have a higher specific surface area and can be expected to show higher reactivity [53]. Dahle et al. (2015) observed that phosphate absorption gradually decreased with increasing pH for small CeO_2_ NPs, and larger NPs had a pH independent behaviour. Large CeO_2_ NPs contained more exchangeable Ce^3+^ than smaller NPs, which could mean that the exchangeable Ce^3+^ facilitated the precipitation of CePO_4_ at the CeO_2_–water interface [19].

Particle shape could have also influenced the reaction in the case of Ce212, which were the only cubic shaped particles, while the others were mostly spherical. Zhang et al. (2017) found that rod-like CeO_2_ NPs had the highest chemical reactivity towards phosphate compared to octahedral, cubic and irregularly shaped NPs in hydroponic cucumber plant media similar to the one used in this study [54].

### UV-vis and zeta potential

**Fig 4:**
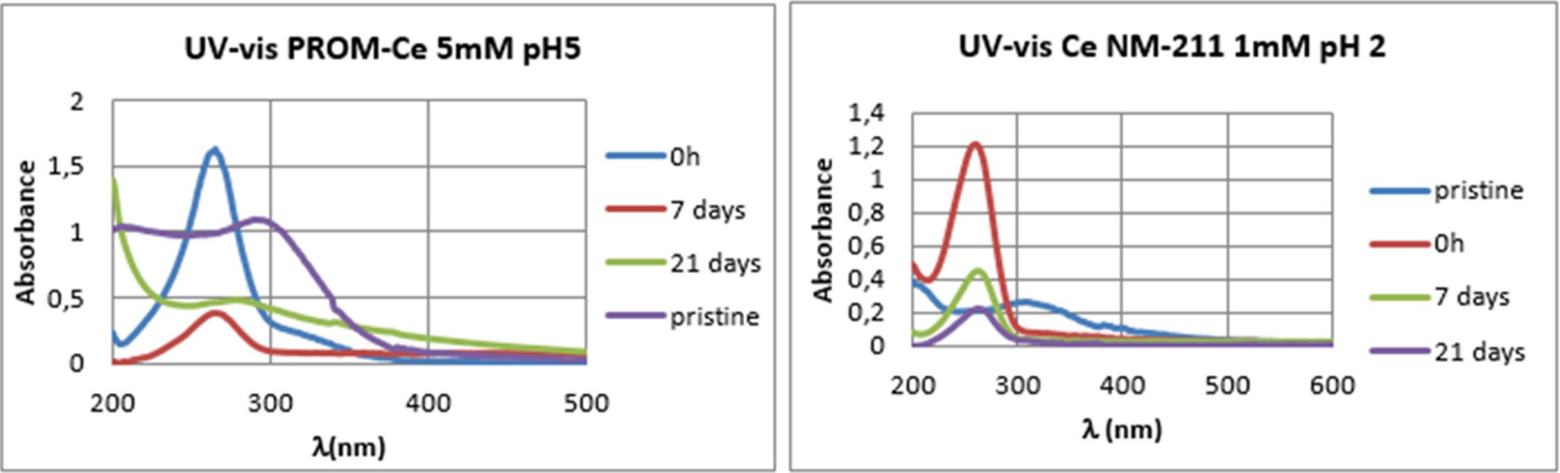
UV-vis spectra. UV-vis spectra for a complete reaction (PROM-Ce 5mM pH5) and an incomplete reaction (Ce NM-211 1mM pH2).

The particle’s UV absorbance was measured at the beginning of the exposure, after 7 days and after 21 days, an example of a successful and an unsuccessful transformation to CePO_4_ are shown in Fig 4. At time zero, a large peak at around 260 nm can be observed, which corresponds to the absorption spectrum of ascorbic acid [55], and in some cases can still be observed after 7 days (observed for Ce NM-211 1mM pH2 and 5, and 5mM pH2; for Ce NM-212 1mM pH2 and 5 mM pH 2 and 5, and for PROM-Ce 5mM pH2 and 5). The ascorbic acid reducing the CeO_2_ NPs becomes oxidised to dehydroascorbic acid which does not show a peak in the recorded range [56]. At 21 days a spectrum that differed from the pristine particles can be observed where the reaction could have been complete. The peaks for Ce^3+^ can be observed at 200 nm and for Ce^4+^ at ~ 300–320 nm, and in the case of the aged particles a clear increase in the Ce^3+^ can be observed compared to the pristine particles. Reactions that had a different spectrum after 21 days were Ce NM-211 1mM pH 12 and 5 mM pH 5 and 12, and Ce NM-212, PROM-Ce and Ce10 at 1mM and 5mM at all pH values. An ascorbic acid peak could still be observed after 21 days for some of the particles where the transformation was not successful (Ce NM-211 1 mM pH 2, shown in Fig 4, and pH 5, and 5mM pH 2), possibly implying that the first step of the transformation was not successful, the dissolution with citric acid.

**Fig 5:**
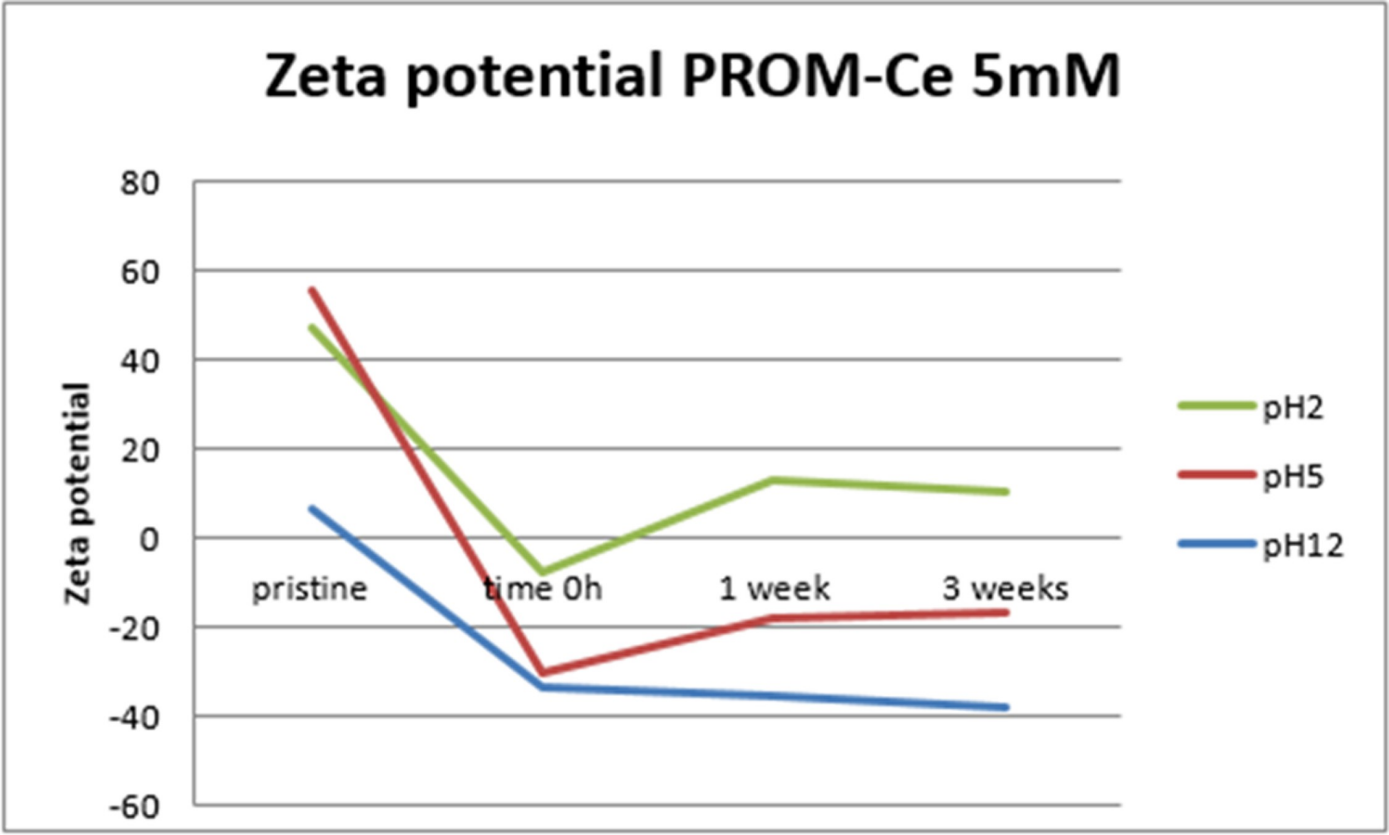
Zeta potential obtained for PROM-Ce 5mM. The graph shows results at different pH values and different times.

Fig 5 shows the zeta potential results measured for PROM-Ce at 5 mM and different pH. No trend was observed in any case, but we found that at 21 days the final zeta potential value seems to be negative (S15–21 Figs), except for most particles at pH 2. Our results agree with Cornelis et al. (2011) and McCormack et al. (2014), where they found that surface adsorption of phosphate to CeO_2_ NPs caused a negative zeta potential [57,58]. McCormack et al. (2014) stated that the phosphate ion concentration has a direct effect on the NP zeta potential by modifying the outer Helmholtz plane and compressing the double layer as the ions concentration increases [58].

### XPS

**Fig 6:**
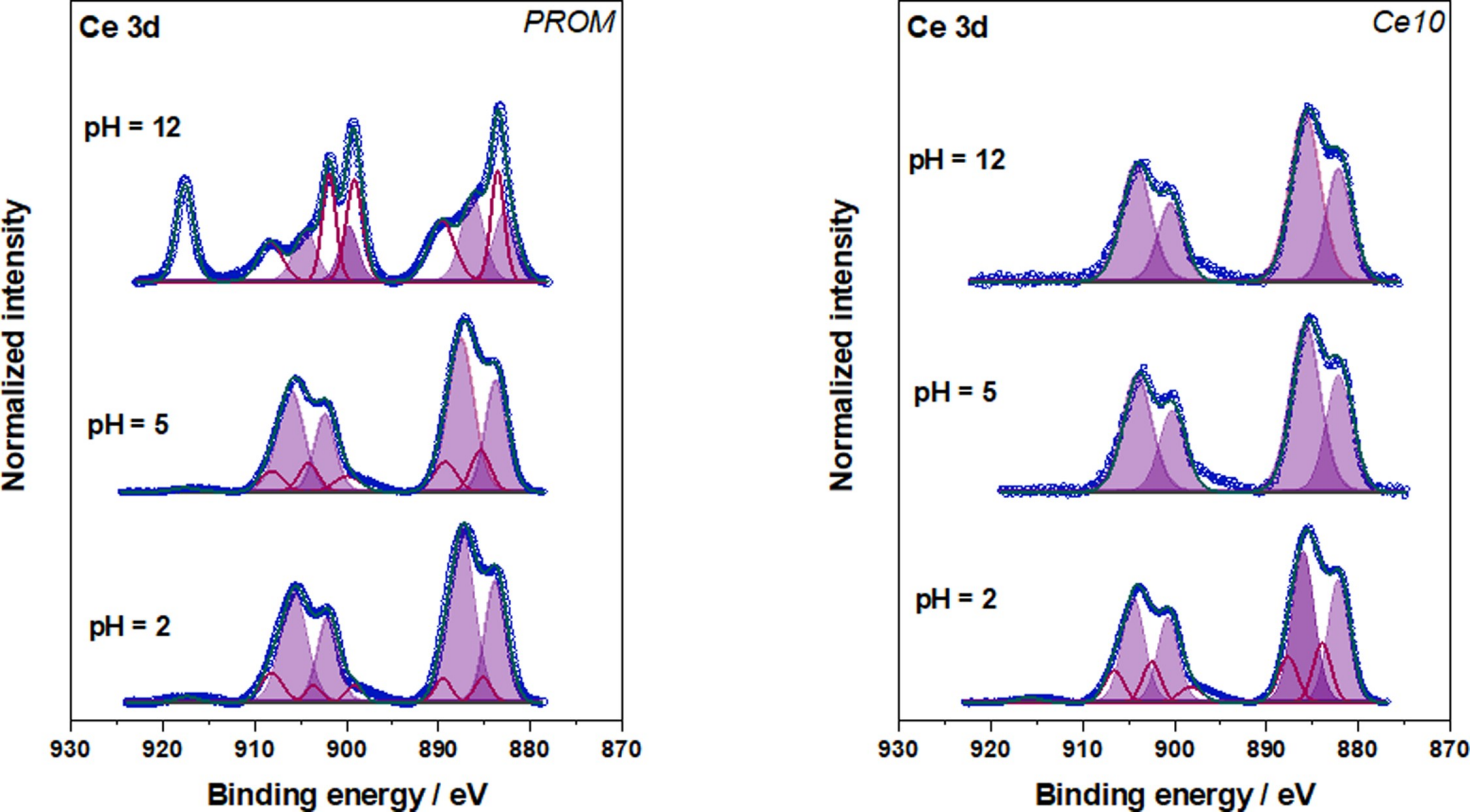
Ce 3d XP spectra for the PROM-Ce samples (left) and Ce10 samples (right) at 5mM and different pH values. The violet filled peaks belong to Ce^3+^ whereas the red ones and the peak at ~917 eV belong to Ce^4+^.

The oxidation state of the Ce was determined via XPS for the PROM-Ce and Ce-10 samples at 5mM and all pH values after 21 days (Fig 6). For the Ce211 and Ce212 samples, only a partial characterisation could be achieved and the inconclusive results are not shown here. Bêche et al. (2008) reported that Ce^3+^ shows two doublets composed of peaks at 880.9, 885.0, 899.1 and 903.5 eV whilst the multiplet structure of Ce^4+^ is composed of 6 peaks at 882.1, 888.1, 898.0, 900.9, 906.4, and 916.4 eV [34,59,60]. The satellite peak at 916.7 eV is characteristic of Ce^4+^and well separated from all other ones^.^ [61].

According to the results obtained, 5mM PROM-Ce at pH 2 and 5 contained predominantly Ce^3+^ (violet peaks), whereas at pH 12 Ce was present as a mixture of +3 (violet peaks) and +4, clearly shown by the peak at ~ 917 eV, together with the multiplet (red peaks) [60]. In the case of Ce10 NPs at 5mM and pH 2, almost exclusively Ce^3+^ was present. In the case of pH5 and 12, only Ce^3+^ was evidenced, which supports the observations made with EDX.

## Conclusions

We have studied the ageing of different CeO_2_ NPs under different conditions mimicking exposure to environments where phosphate and a range of pH may be occurring. We found that concentration of the reagents, particle size, oxidation state, capping agent, the presence of organic acids and reducing agent, and pH all have an effect on the formation of CePO_4_ particles. All CeO_2_ NPs transformed to CePO_4_ at 5mM reagent concentration and pH 5. At 1mM concentration and pH 5 the reaction was only complete for PROM-Ce and Ce10. It has been observed that for some plant species (e.g., corn and wheat), deficiency of P can increase the potential phytotoxicity of CeO_2_ NPs and enhance the accumulation of Ce (mainly in the form of Ce^3+^) in plants [40,62]. The particle’s oxidation state played a key role in the dissolution of the CeO_2_ NPs and the formation of CePO_4_, thus Ce NM-211 and Ce NM-212, which contained mainly Ce^4+^ and were therefore already fully oxidised, did not dissolve at 1mM pH 5, whereas PROM-Ce and Ce10 (which were a mix of Ce^3+^ and Ce^4+^) did. In the case of Ce10 we think that the PVP had an additional reducing effect, thus permitting a more efficient formation of CePO_4_ particles at every condition used. CeO_2_ NPs in the environment could follow the type of transformations observed in this work, thus it is very important to assess how NMs transform during ecotoxicological assays. Further studies are needed in environmentally relevant conditions, such as natural and waste waters or soils where natural organic matter is present.

## Supporting information

S1 Fig Speciation of PO_4_^3-^ ions. Expressed as mole fraction of total P and in solution as a function of pH.
(TIFF)

S2 Fig STEM and TEM images obtained for Ce NM-211. Images obtained for pristine Ce NM-211 (STEM) and at different concentrations of phosphate and at different pH after 21 days (TEM).
(TIFF)

S3 Fig STEM and TEM images obtained for Ce NM-212. Obtained for pristine Ce NM-212 (STEM) and at different concentrations of phosphate and at different pH after 21 days (TEM).
(TIFF)

S4 Fig STEM and TEM images obtained for PROM-Ce. Images for pristine PROM-Ce (STEM) and at different concentrations of phosphate and at different pH after 21 days (TEM).
(TIFF)

S5 Fig EDX results obtained for Ce NM-211 at 1mM and pH 5 after 21 days of exposure. It can be observed that no P peak was found.
(TIFF)

S6 Fig EDX results obtained for Ce NM-212 at 1mM and pH 5 after 21 days of exposure. It can be observed that no P peak was found.
(TIFF)

S7 Fig EDX results obtained for PROM-Ce at 1mM and pH 5 after 21 days of exposure. It can be observed that a P peak was found.
(TIFF)

S8 Fig EDX results obtained for Ce10 at 1mM and pH 5 after 21 days of exposure. It can be observed that a P peak was found.
(TIFF)

S9 Fig EDX results obtained for Ce10 at 5mM and pH 2 after 21 days of exposure. It can be observed that a P peak was found.
(TIFF)

S10 Fig EDX results obtained for Ce10 at 5mM and pH 5 after 21 days of exposure. It can be observed that a P peak was found and “sea urchin” structures were observed.
(TIFF)

S11 Fig EDX results obtained for Ce10 at 5mM and pH 12 after 21 days of exposure. It can be observed that a P peak was found.
(TIFF)

S12 Fig EDX point spectrum results obtained for PROM-Ce at 5 mM and pH 5 after 21 days of exposure. It can be observed that a P peak was found when the “sea urchin” structure was measured (spectrum 1).
(TIFF)

S13 Fig EDX map obtained for PROM-Ce at 5 mM and pH 5 after 21 days of exposure. It can be observed that a Ce (green) and a P (red) signal were found throughout the “sea urchin” structures.
(TIFF)

S14 Fig STEM-EELS spectra acquired on different spots of the same PROM-Ce nanoparticle. We observed a co-existence of Ce^3+^ and Ce^4+^ valence states for each nanoparticle.
(TIFF)

S15 Fig Zeta potential values for Ce NM-211 at 1mM. Different pH values and different exposure time points are shown.
(TIFF)

S16 Fig Zeta potential values for Ce NM-212 at 1mM. Different pH values and different exposure time points are shown.
(TIFF)

S17 Fig Zeta potential values for PROM-Ce at 1mM. Different pH values and different exposure time points are shown.
(TIFF)

S18 Fig Zeta potential values for Ce10 at 1mM. Different pH values and different exposure time points are shown.
(TIFF)

S19 Fig Zeta potential values for Ce NM-211 at 5mM. Different pH values and different exposure time points are shown.
(TIFF)

S20 Fig Zeta potential values for Ce NM-212 at 5mM. Different pH values and different exposure time points are shown.
(TIFF)

S21 Fig Zeta potential values for Ce10 at 5mM. Different pH values and different exposure time points are shown.
(TIFF)

S1 Data Raw data zip file. Includes the raw data used for all graphs presented.
(ZIP)

## References

[1] Nowack B, Bucheli TD. Occurrence, behavior and effects of nanoparticles in the environment. Environ Pollut. Elsevier; 2007;150: 5–22. 10.1016/j.envpol.2007.06.006 17658673

[2] Merrifield RC, Wang ZW, Palmer RE, Lead JR. Synthesis and characterization of polyvinylpyrrolidone coated cerium oxide nanoparticles. Environ Sci Technol. ACS Publications; 2013;47: 12426–12433. 10.1021/es402541z 24044591

[3] Römer I, Wang ZW, Merrifield RC, Palmer RE, Lead J. High Resolution STEM-EELS Study of Silver Nanoparticles Exposed to Light and Humic Substances. Environ Sci Technol. 2016;50 10.1021/acs.est.5b04088 26792384

[4] REACH. Second Regulatory Review on Nanomaterials [Internet]. 2012. Available: http://eur-lex.europa.eu/legal-content/EN/TXT/?uri=CELEX:52012DC0572

[5] Montaño MD, Lowry G V, von der Kammer F, Blue J, Ranville JF. Current status and future direction for examining engineered nanoparticles in natural systems. Environ Chem. CSIRO; 2014;11: 351–366.

[6] Vance ME, Kuiken T, Vejerano EP, McGinnis SP, Hochella MF, Hull DR. Nanotechnology in the real world: Redeveloping the nanomaterial consumer products inventory. Beilstein J Nanotechnol. 2015;6: 1769–1780. 10.3762/bjnano.6.181 26425429 PMC4578396

[7] Valsami-Jones E, Lynch I. How safe are nanomaterials? Science (80-). American Association for the Advancement of Science; 2015;350: 388–389. 10.1126/science.aad0768 26494749

[8] Stanek CR, Tan AHH, Owens SL, Grimes RW. Atomistic simulation of CeO2 surface hydroxylation: implications for glass polishing. J Mater Sci. Springer; 2008;43: 4157–4162.

[9] Hajjari M, Ardjmand M, Tabatabaei M. Experimental investigation of the effect of cerium oxide nanoparticles as a combustion-improving additive on biodiesel oxidative stability: mechanism. RSC Adv. Royal Society of Chemistry; 2014;4: 14352–14356.

[10] Reed K, Cormack A, Kulkarni A, Mayton M, Sayle D, Klaessig F, et al Exploring the properties and applications of nanoceria: is there still plenty of room at the bottom? Environ Sci Nano. Royal Society of Chemistry; 2014;1: 390–405.

[11] Marchiol L, Mattiello A, Pošćić F, Fellet G, Zavalloni C, Carlino E, et al Changes in physiological and agronomical parameters of barley (Hordeum vulgare) exposed to cerium and titanium dioxide nanoparticles. Int J Environ Res Public Health. Multidisciplinary Digital Publishing Institute; 2016;13: 332. 10.3390/ijerph13030332 PMC4808995 26999181

[12] Schwabe F, Schulin R, Rupper P, Rotzetter A, Stark W, Nowack B. Dissolution and transformation of cerium oxide nanoparticles in plant growth media. J nanoparticle Res. Springer; 2014;16: 2668.

[13] Schwab F, Zhai G, Kern M, Turner A, Schnoor JL, Wiesner MR. Barriers, pathways and processes for uptake, translocation and accumulation of nanomaterials in plants–Critical review. Nanotoxicology. Taylor & Francis; 2016;10: 257–278. 10.3109/17435390.2015.1048326 26067571

[14] Zhang P, Ma Y, Zhang Z, He X, Zhang J, Guo Z, et al Biotransformation of ceria nanoparticles in cucumber plants. ACS Nano. ACS Publications; 2012;6: 9943–9950. 10.1021/nn303543n 23098040

[15] Baalousha M, Le Coustumer P, Jones I, Lead JR. Characterisation of structural and surface speciation of representative commercially available cerium oxide nanoparticles. Environ Chem. CSIRO; 2010;7: 377–385.

[16] Dekkers S, Miller MR, Schins RPF, Römer I, Russ M, Vandebriel RJ, et al The effect of zirconium doping of cerium dioxide nanoparticles on pulmonary and cardiovascular toxicity and biodistribution in mice after inhalation. Nanotoxicology. 2017;11: 794–808. 10.1080/17435390.2017.1357214 28741972

[17] Trovarelli A, Boaro M, Rocchini E, de Leitenburg C, Dolcetti G. Some recent developments in the characterization of ceria-based catalysts. J Alloys Compd. Elsevier; 2001;323: 584–591.

[18] Beaudoux X, Virot M, Chave T, Leturcq G, Clavier N, Dacheux N, et al Catalytic dissolution of ceria–lanthanide mixed oxides provides environmentally friendly partitioning of lanthanides and platinum. Hydrometallurgy. Elsevier; 2015;151: 107–115.

[19] Dahle JT, Livi K, Arai Y. Effects of pH and phosphate on CeO2 nanoparticle dissolution. Chemosphere. 2015;119: 1365–1371. 10.1016/j.chemosphere.2014.02.027 24630459

[20] Gaiser BK, Fernandes TF, Jepson M a, Lead JR, Tyler CR, Baalousha M, et al Interspecies comparisons on the uptake and toxicity of silver and cerium dioxide nanoparticles. Environ Toxicol Chem. 2012;31: 144–154. 10.1002/etc.703 22002553

[21] Baalousha M, Ju-Nam Y, Cole PA, Gaiser B, Fernandes TF, Hriljac JA, et al Characterization of cerium oxide nanoparticles—part 1: size measurements. Environ Toxicol Chem. Wiley Online Library; 2012;31: 983–993. 10.1002/etc.1785 22368045

[22] Badawy AM El, Luxton TP, Silva RG, Scheckel KG, Suidan MT, Tolaymat TM. Impact of environmental conditions (pH, ionic strength, and electrolyte type) on the surface charge and aggregation of silver nanoparticles suspensions. Environ Sci Technol. ACS Publications; 2010;44: 1260–1266. 10.1021/es902240k 20099802

[23] Adschiri T, Lee Y-W, Goto M, Takami S. Green materials synthesis with supercritical water. Green Chem. Royal Society of Chemistry; 2011;13: 1380–1390.

[24] Charbgoo F, Bin Ahmad M, Darroudi M. Cerium oxide nanoparticles: green synthesis and biological applications. Int J Nanomedicine. Dove Press; 2017;12: 1401 10.2147/IJN.S124855 28260887 PMC5325136

[25] Virkutyte J, Varma RS. Green synthesis of metal nanoparticles: biodegradable polymers and enzymes in stabilization and surface functionalization. Chem Sci. Royal Society of Chemistry; 2011;2: 837–846.

[26] Meiser F, Cortez C, Caruso F. Biofunctionalization of fluorescent rare-earth-doped lanthanum phosphate colloidal nanoparticles. Angew Chemie Int Ed. Wiley Online Library; 2004;43: 5954–5957. 10.1002/anie.200460856 15547904

[27] Li G, Chao K, Peng H, Chen K, Zhang Z. Facile synthesis of CePO4 nanowires attached to CeO2 octahedral micrometer crystals and their enhanced photoluminescence properties. J Phys Chem C. ACS Publications; 2008;112: 16452–16456.

[28] Pirmohamed T, Dowding JM, Singh S, Wasserman B, Heckert E, Karakoti AS, et al Nanoceria exhibit redox state-dependent catalase mimetic activity. Chem Commun (Camb). 2010;46: 2736–2738. 10.1039/b922024k 20369166 PMC3038687

[29] Xue Y, Zhai Y, Zhou K, Wang L, Tan H, Luan Q, et al The vital role of buffer anions in the antioxidant activity of CeO2 nanoparticles. Chem Eur J. Wiley Online Library; 2012;18: 11115–11122. 10.1002/chem.201200983 22807390

[30] Nelson BC, Johnson ME, Walker ML, Riley KR, Sims CM. Antioxidant Cerium Oxide Nanoparticles in Biology and Medicine Reipa V, editor. Antioxidants. MDPI; 2016;5: 15 10.3390/antiox5020015 PMC4931536 27196936

[31] Naganuma T, Traversa E. The effect of cerium valence states at cerium oxide nanoparticle surfaces on cell proliferation. Biomaterials. 2014;35: 4441–4453. 10.1016/j.biomaterials.2014.01.074 24612920

[32] Singh C, Friedrichs S, Ceccone G, Gibson N, Jensen KA, Levin M, et al. Cerium Dioxide, NM-211, NM-212, NM-213. Characterisation and test item preparation. EUR-scientific Tech Res reports Luxemb JRC ICHP, EUR. 2014;26649.

[33] Promethean_Particles. Dispersion catalogues [Internet]. Available: http://www.prometheanparticles.co.uk/wp-content/uploads/2017/04/Promethean-Particles-Dispersion-portolio-2017.pdf

[34] Briffa SM, Lynch I, Trouillet V, Bruns M, Hapiuk D, Liu J, et al Development of scalable and versatile nanomaterial libraries for nanosafety studies: polyvinylpyrrolidone (PVP) capped metal oxide nanoparticles. RSC Adv. Royal Society of Chemistry; 2017;7: 3894–3906.

[35] Trejo-Téllez LI, Gómez-Merino FC. Nutrient solutions for hydroponic systems Hydroponics-A Standard Methodology for Plant Biological Researches. InTech; 2012.

[36] Lissner J, Mendelssohn IA, Anastasiou CJ. A method for cultivating plants under controlled redox intensities in hydroponics. Aquat Bot. Elsevier; 2003;76: 93–108.

[37] Lowry G V, Gregory KB, Apte SC, Lead JR. Transformations of Nanomaterials in the Environment. Environ Sci Technol. 2012;46: 6893–6899. 10.1021/es300839e 22582927

[38] Jensen KA, Kembouche Y, Christiansen E, Jacobsen NR, Wallin H, Guiot C, et al Final protocol for producing suitable manufactured nanomaterial exposure media. NANOGENOTOX Deliv Rep n. 2011;3.

[39] Cabanas A, Darr JA, Lester E, Poliakoff M. A continuous and clean one-step synthesis of nano-particulate Ce1− xZrxO2 solid solutions in near-critical water. Chem Commun. Royal Society of Chemistry; 2000; 901–902.

[40] Wang G, Ma Y, Zhang P, He X, Zhang Z, Qu M, et al Influence of phosphate on phytotoxicity of ceria nanoparticles in an agar medium. Environ Pollut. Elsevier; 2017;224: 392–399. 10.1016/j.envpol.2017.02.019 28237306

[41] Römer I, Gavin AJ, White TA, Merrifield RC, Chipman JK, Viant MR, et al The critical importance of defined media conditions in Daphnia magna nanotoxicity studies. Toxicol Lett. 2013;223 10.1016/j.toxlet.2013.08.026 24021169

[42] Parry KL, Shard AG, Short RD, White RG, Whittle JD, Wright A. ARXPS characterisation of plasma polymerised surface chemical gradients. Surf Interface Anal An Int J devoted to Dev Appl Tech Anal surfaces, interfaces thin Film. Wiley Online Library; 2006;38: 1497–1504.

[43] Scofield JH. Hartree-Slater subshell photoionization cross-sections at 1254 and 1487 eV. J Electron Spectros Relat Phenomena. Elsevier; 1976;8: 129–137.

[44] Lawrence NJ, Jiang K, Cheung CL. Formation of a porous cerium oxide membrane by anodization. Chem Commun. Royal Society of Chemistry; 2011;47: 2703–2705. 10.1039/c0cc04806b 21234482

[45] Jalilpour M, Fathalilou M. Effect of aging time and calcination temperature on the cerium oxide nanoparticles synthesis via reverse co-precipitation method. Int J Phys Sci. Academic Journals; 2012;7: 944–948.

[46] Verma S, Bamzai KK. Preparation of cerium orthophosphate nanosphere by coprecipitation route and its structural, thermal, optical, and electrical characterization. J Nanoparticles. Hindawi; 2014;2014.

[47] Ingham B. X-ray scattering characterisation of nanoparticles. Crystallogr Rev. Taylor & Francis; 2015;21: 229–303.

[48] Li R, Ji Z, Chang CH, Dunphy DR, Cai X, Meng H, et al Surface interactions with compartmentalized cellular phosphates explain rare earth oxide nanoparticle hazard and provide opportunities for safer design. ACS Nano. ACS Publications; 2014;8: 1771–1783. 10.1021/nn406166n 24417322 PMC3988685

[49] Mirshafiee V, Sun B, Chang CH, Liao Y, Jiang W, Jiang J, et al Toxicological Profiling of Metal Oxide Nanoparticles in Liver Context Reveals Pyroptosis in Kupffer Cells and Macrophages versus Apoptosis in Hepatocytes. ACS Nano. 2018;12: 3836–3852. 10.1021/acsnano.8b01086 29543433 PMC5946698

[50] Koczkur KM, Mourdikoudis S, Polavarapu L, Skrabalak SE. Polyvinylpyrrolidone (PVP) in nanoparticle synthesis. Dalt Trans. Royal Society of Chemistry; 2015;44: 17883–17905. 10.1039/c5dt02964c 26434727

[51] Ma Y, He X, Zhang P, Zhang Z, Guo Z, Tai R, et al Phytotoxicity and biotransformation of La2O3 nanoparticles in a terrestrial plant cucumber (Cucumis sativus). Nanotoxicology. Taylor & Francis; 2011;5: 743–753. 10.3109/17435390.2010.545487 21261455

[52] Schwabe F, Tanner S, Schulin R, Rotzetter A, Stark W, von Quadt A, et al Dissolved cerium contributes to uptake of Ce in the presence of differently sized CeO2-nanoparticles by three crop plants. Metallomics. The Royal Society of Chemistry; 2015;7: 466–477. 10.1039/c4mt00343h 25634091

[53] Gui X, Zhang Z, Liu S, Ma Y, Zhang P, He X, et al Fate and phytotoxicity of CeO2 nanoparticles on lettuce cultured in the potting soil environment. PLoS One. Public Library of Science; 2015;10: e0134261 10.1371/journal.pone.0134261 26317617 PMC4552829

[54] Zhang P, Xie C, Ma Y, He X, Zhang Z, Ding Y, et al Shape-Dependent Transformation and Translocation of Ceria Nanoparticles in Cucumber Plants. Environ Sci Technol Lett. American Chemical Society; 2017;4: 380–385. 10.1021/acs.estlett.7b00359

[55] Tóth M, Kukor Z, Valent S. Chemical stabilization of tetrahydrobiopterin by L-ascorbic acid: contribution to placental endothelial nitric oxide synthase activity. MHR Basic Sci Reprod Med. European Society of Human Reproduction and Embryology; 2002;8: 271–280. 10.1093/molehr/8.3.271 11870235

[56] Root-Bernstein R, Fewins J, Rhinesmith T, Koch A, Dillon PF. Enzymatic recycling of ascorbic acid from dehydroascorbic acid by glutathione-like peptides in the extracellular loops of aminergic G-protein coupled receptors. J Mol Recognit. Wiley Online Library; 2016;29: 296–302. 10.1002/jmr.2530 26749062

[57] Cornelis G, Ryan B, McLaughlin MJ, Kirby JK, Beak D, Chittleborough D. Solubility and batch retention of CeO2 nanoparticles in soils. Environ Sci Technol. ACS Publications; 2011;45: 2777–2782. 10.1021/es103769k 21405081

[58] McCormack RN, Mendez P, Barkam S, Neal CJ, Das S, Seal S. Inhibition of nanoceria’s catalytic activity due to Ce3+ site-specific interaction with phosphate ions. J Phys Chem C. ACS Publications; 2014;118: 18992–19006.

[59] Kim CK, Kim T, Choi I, Soh M, Kim D, Kim Y, et al Ceria nanoparticles that can protect against ischemic stroke. Angew Chemie Int Ed. Wiley Online Library; 2012;51: 11039–11043. 10.1002/anie.201203780 22968916

[60] Bêche E, Charvin P, Perarnau D, Abanades S, Flamant G. Ce 3d XPS investigation of cerium oxides and mixed cerium oxide (CexTiyOz). Surf Interface Anal. Wiley Online Library; 2008;40: 264–267.

[61] Heckert EG, Karakoti AS, Seal S, Self WT. The role of cerium redox state in the SOD mimetic activity of nanoceria. Biomaterials. Elsevier; 2008;29: 2705–2709. 10.1016/j.biomaterials.2008.03.014 18395249 PMC2396488

[62] Zhang P, Ma Y, Xie C, Guo Z, He X, Valsami-Jones E, et al Plant species-dependent transformation and translocation of ceria nanoparticles. Environ Sci Nano. Royal Society of Chemistry; 2019;

