# Supplementary figures and images for: Impact of particle size, oxidation state and capping agent of different cerium dioxide nanoparticles on the phosphate-induced transformations at different pH and concentration
Source: PLoS One. 2019 Jun 7;14(6):e0217483. doi: 10.1371/journal.pone.0217483 (PMC6555525; doi:10.1371/journal.pone.0217483)

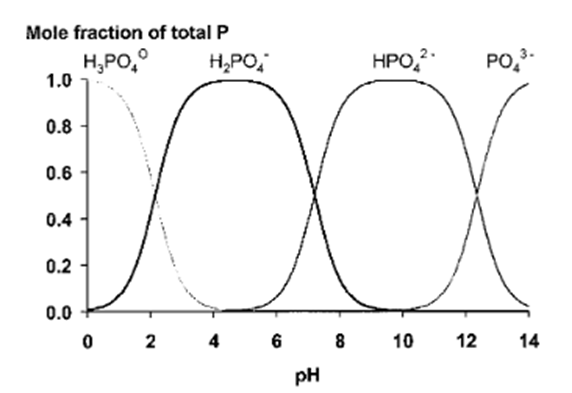

Supplement: S1 Fig — Expressed as mole fraction of total P and in solution as a function of pH. (TIFF) [file pone.0217483.s001.tiff]

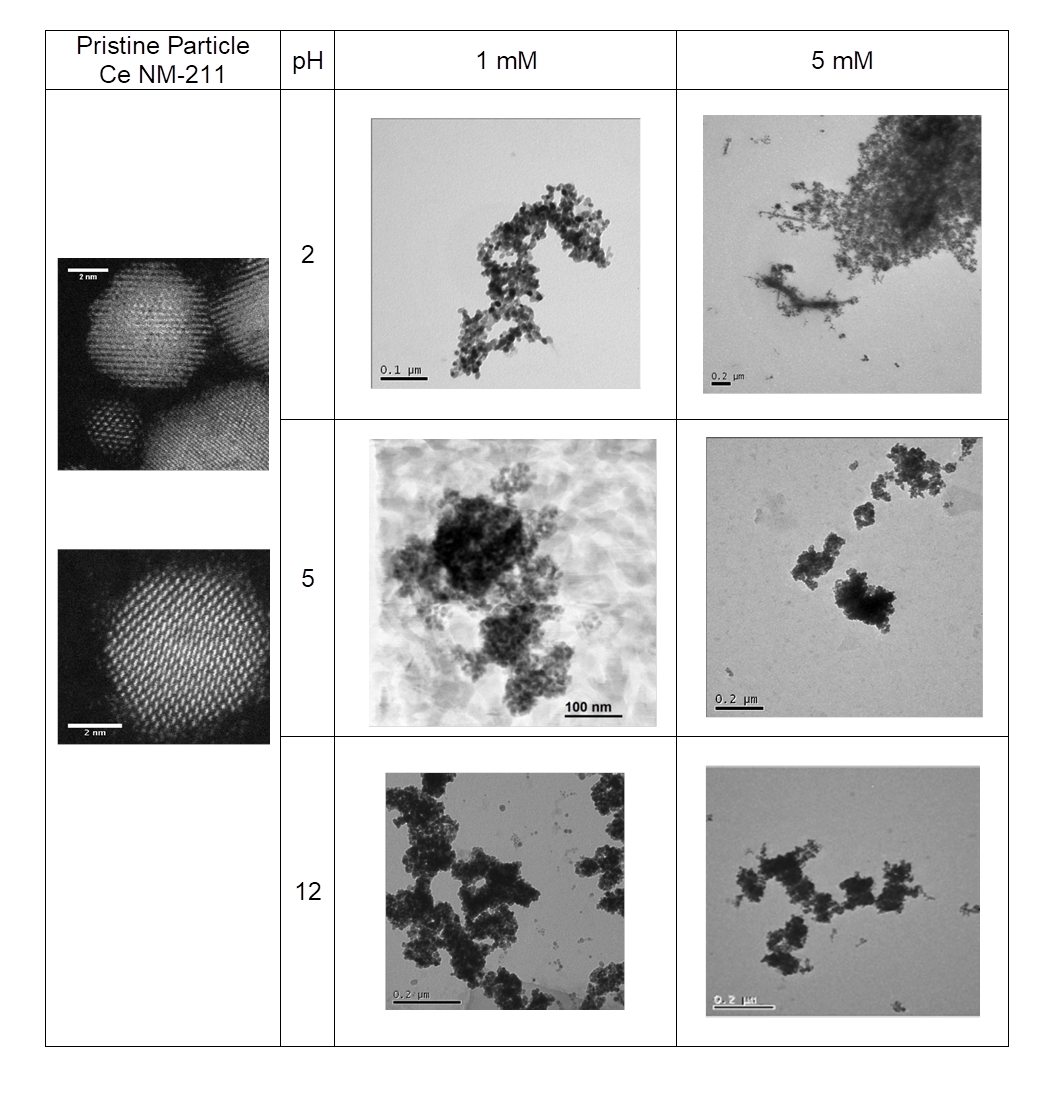

Supplement: S2 Fig — Images obtained for pristine Ce NM-211 (STEM) and at different concentrations of phosphate and at different pH after 21 days (TEM). (TIFF) [file pone.0217483.s002.tiff]

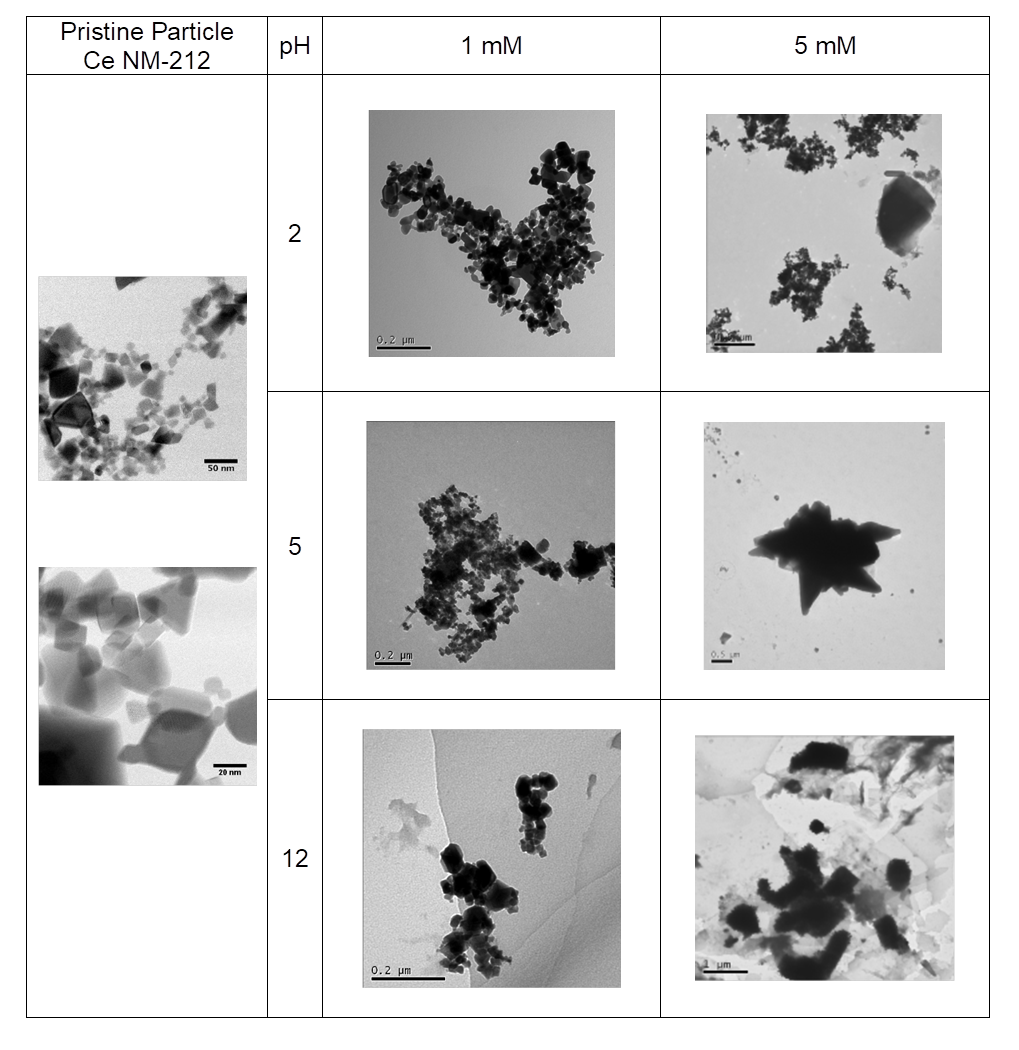

Supplement: S3 Fig — Obtained for pristine Ce NM-212 (STEM) and at different concentrations of phosphate and at different pH after 21 days (TEM). (TIFF) [file pone.0217483.s003.tiff]

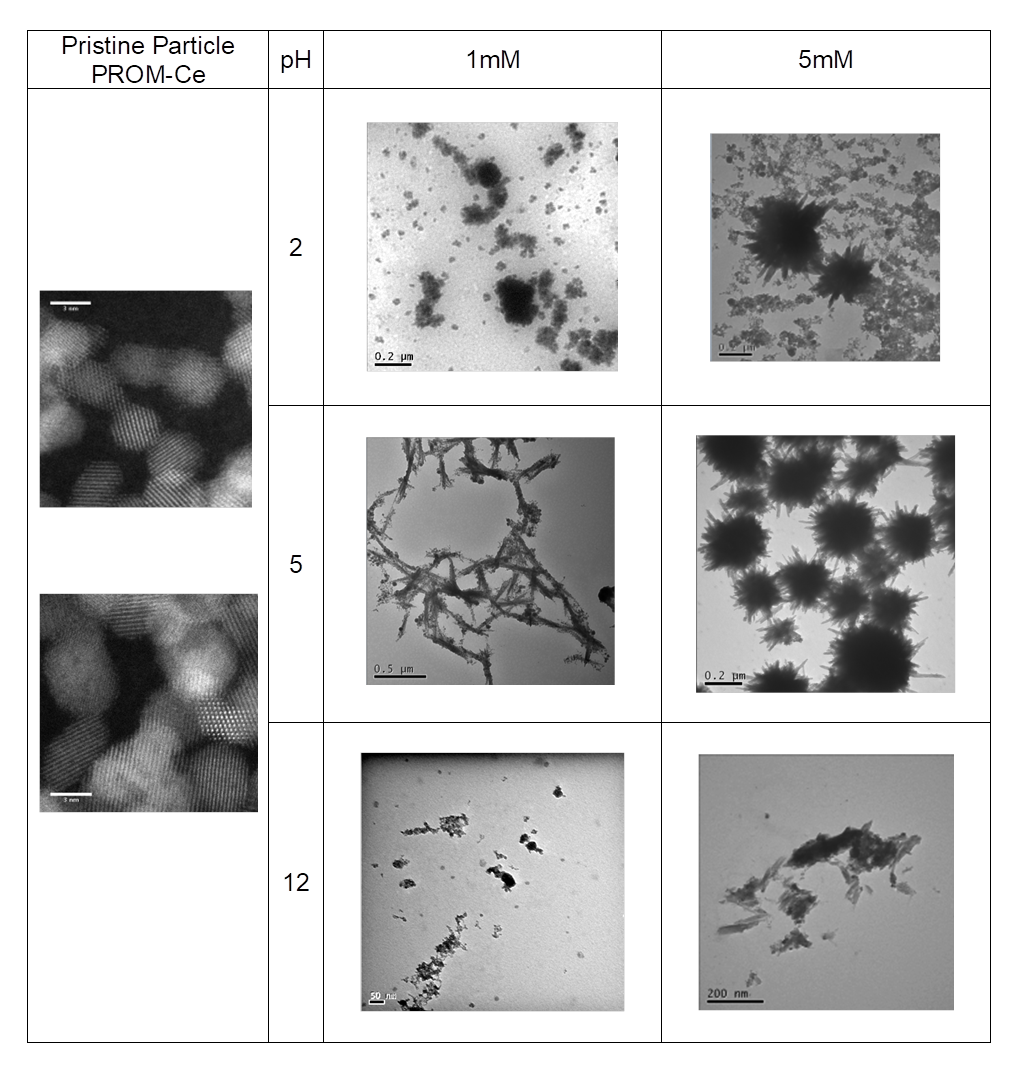

Supplement: S4 Fig — Images for pristine PROM-Ce (STEM) and at different concentrations of phosphate and at different pH after 21 days (TEM). (TIFF) [file pone.0217483.s004.tiff]

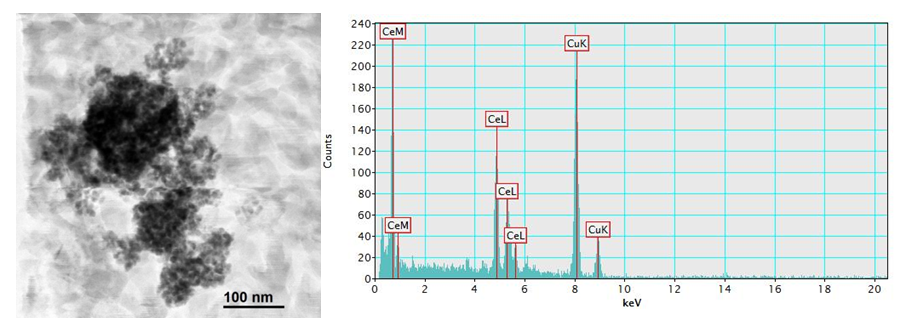

Supplement: S5 Fig — It can be observed that no P peak was found. (TIFF) [file pone.0217483.s005.tiff]

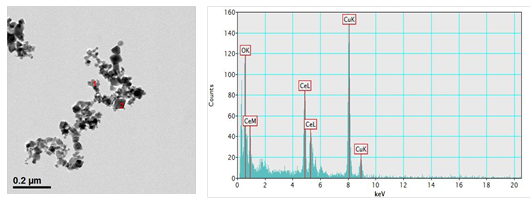

Supplement: S6 Fig — It can be observed that no P peak was found. (TIFF) [file pone.0217483.s006.tiff]

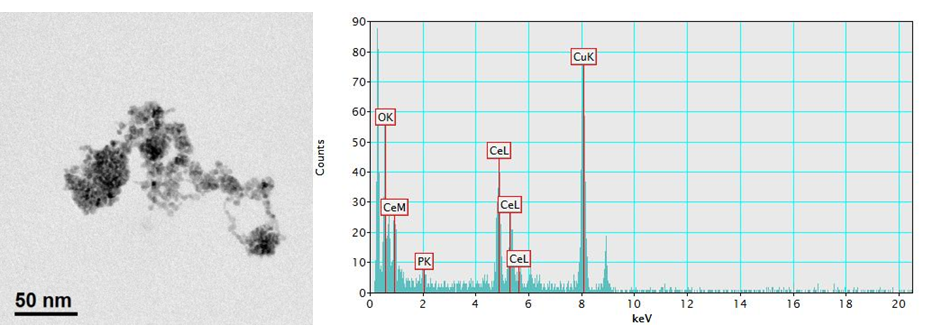

Supplement: S7 Fig — It can be observed that a P peak was found. (TIFF) [file pone.0217483.s007.tiff]

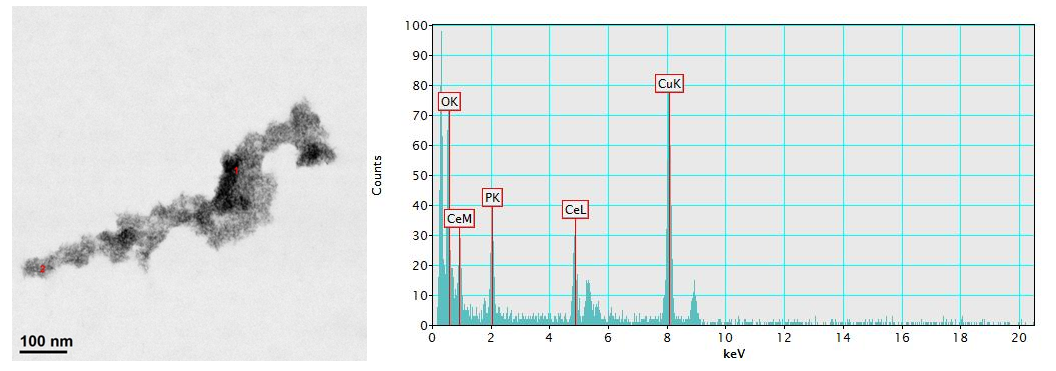

Supplement: S8 Fig — It can be observed that a P peak was found. (TIFF) [file pone.0217483.s008.tiff]

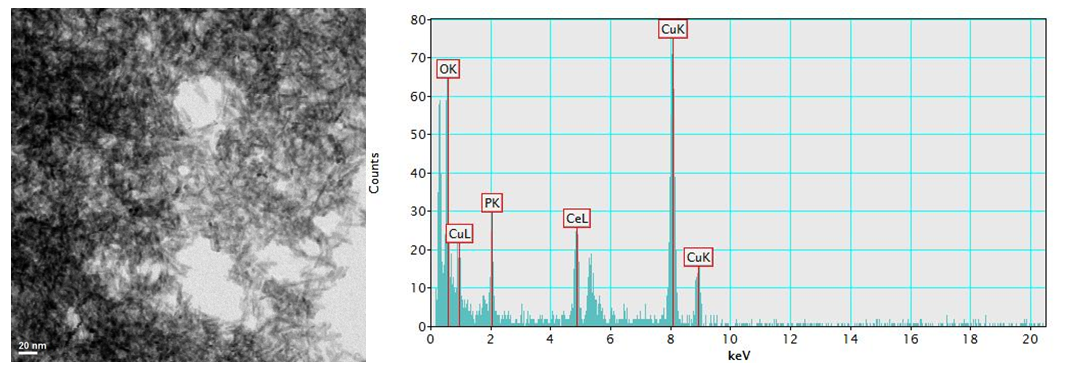

Supplement: S9 Fig — It can be observed that a P peak was found. (TIFF) [file pone.0217483.s009.tiff]

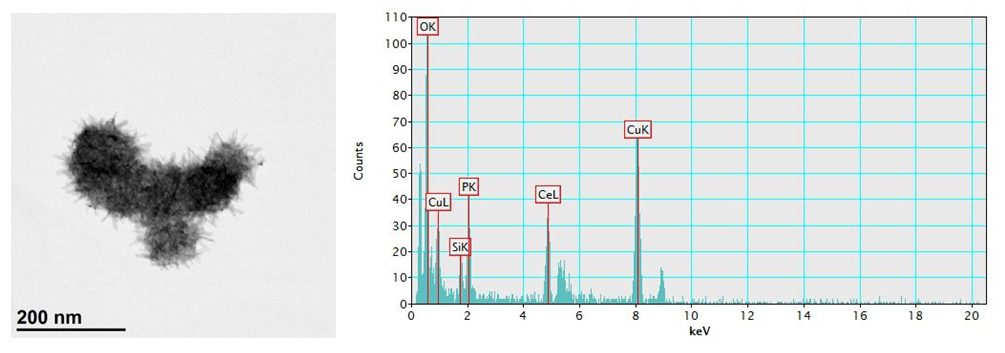

Supplement: S10 Fig — It can be observed that a P peak was found and “sea urchin” structures were observed. (TIFF) [file pone.0217483.s010.tiff]

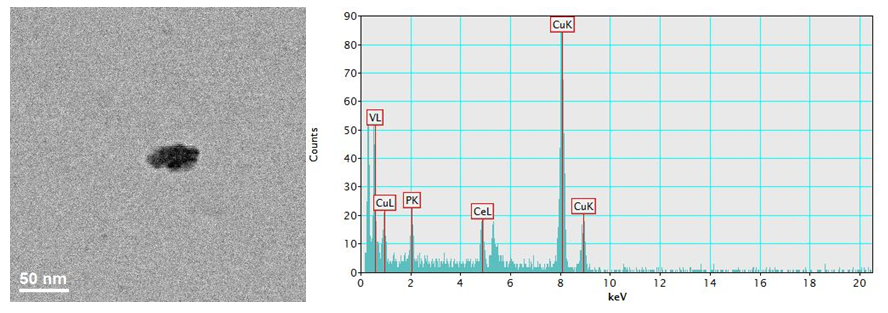

Supplement: S11 Fig — It can be observed that a P peak was found. (TIFF) [file pone.0217483.s011.tiff]

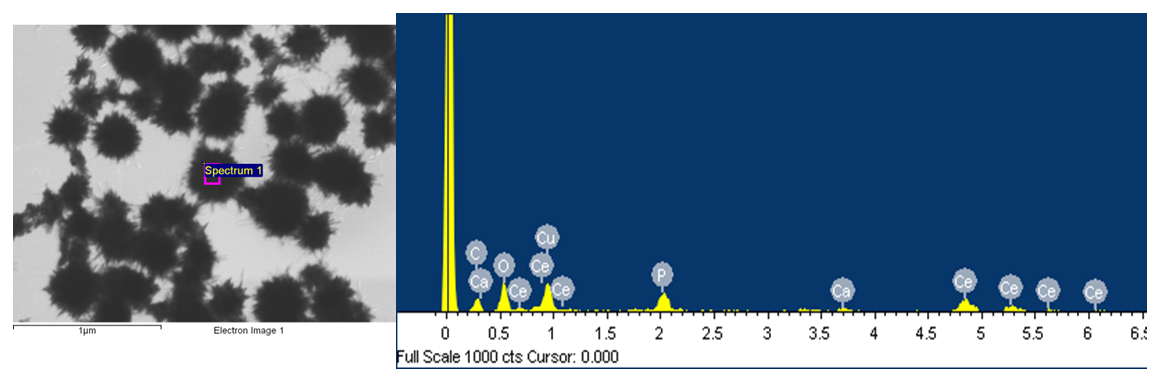

Supplement: S12 Fig — It can be observed that a P peak was found when the “sea urchin” structure was measured (spectrum 1). (TIFF) [file pone.0217483.s012.tiff]

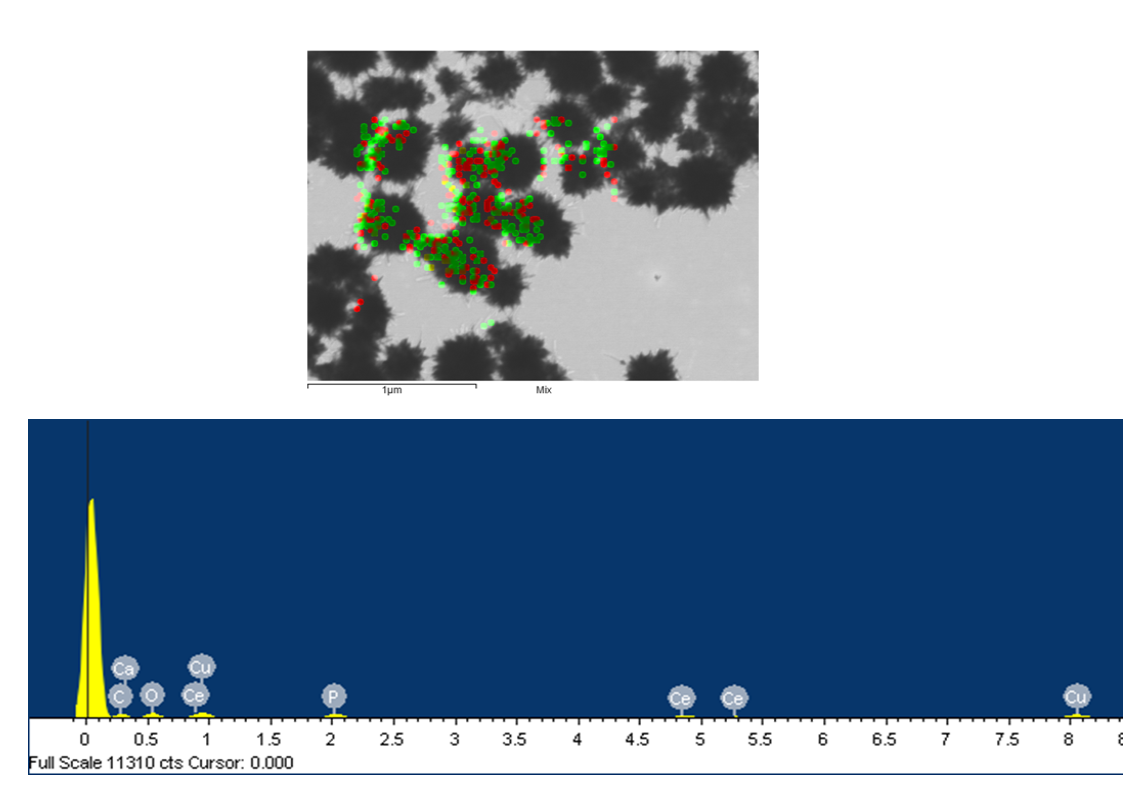

Supplement: S13 Fig — It can be observed that a Ce (green) and a P (red) signal were found throughout the “sea urchin” structures. (TIFF) [file pone.0217483.s013.tiff]

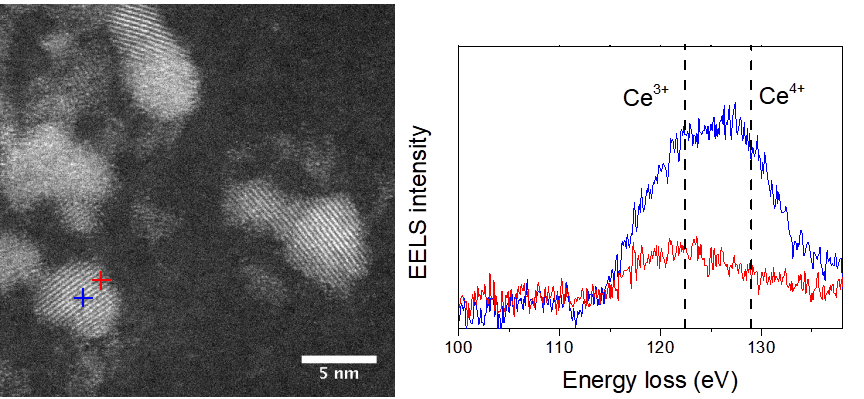

Supplement: S14 Fig — We observed a co-existence of Ce3+ and Ce4+ valence states for each nanoparticle. (TIFF) [file pone.0217483.s014.tiff]

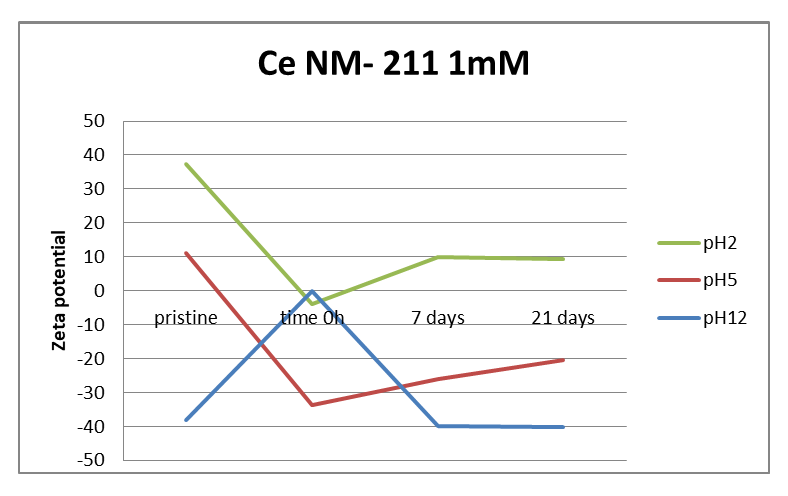

Supplement: S15 Fig — Different pH values and different exposure time points are shown. (TIFF) [file pone.0217483.s015.tiff]

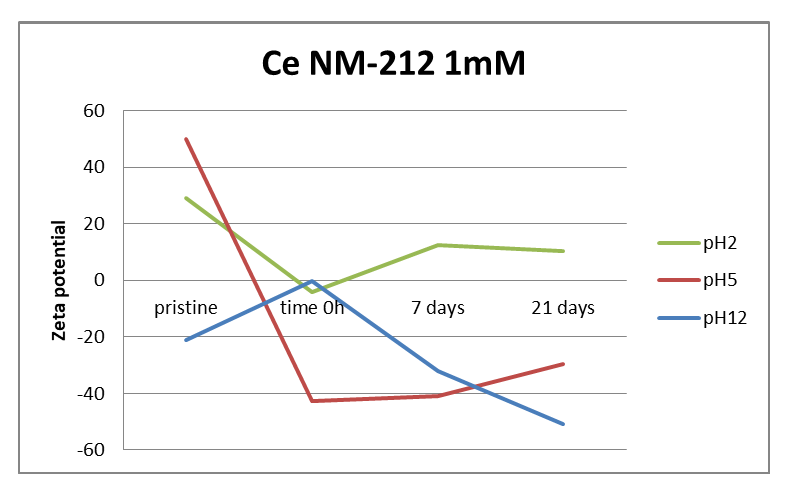

Supplement: S16 Fig — Different pH values and different exposure time points are shown. (TIFF) [file pone.0217483.s016.tiff]

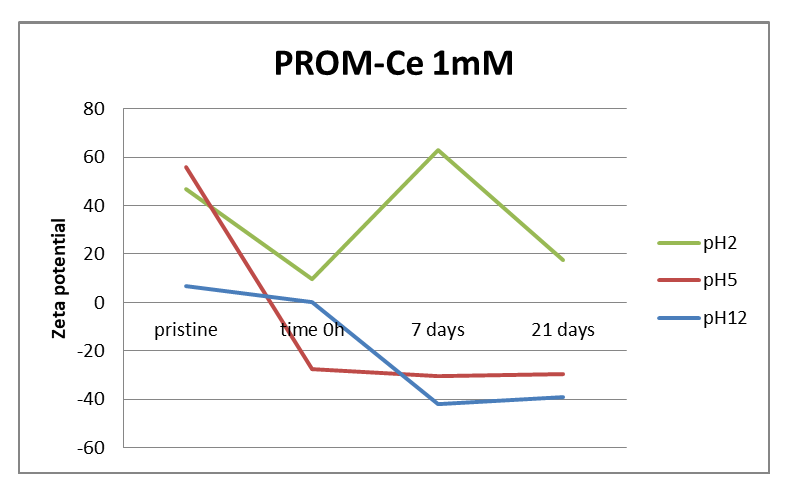

Supplement: S17 Fig — Different pH values and different exposure time points are shown. (TIFF) [file pone.0217483.s017.tiff]

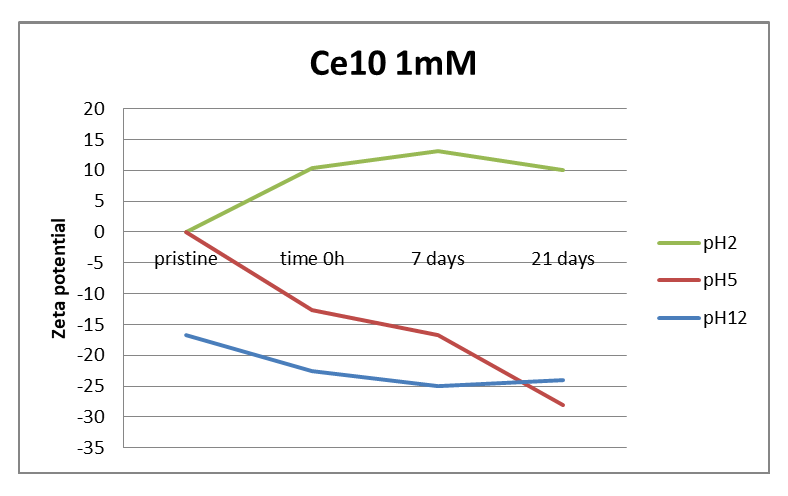

Supplement: S18 Fig — Different pH values and different exposure time points are shown. (TIFF) [file pone.0217483.s018.tiff]

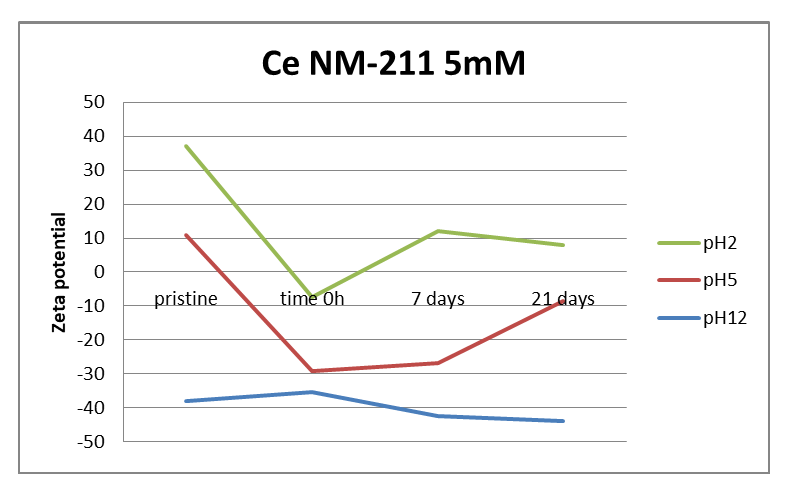

Supplement: S19 Fig — Different pH values and different exposure time points are shown. (TIFF) [file pone.0217483.s019.tiff]

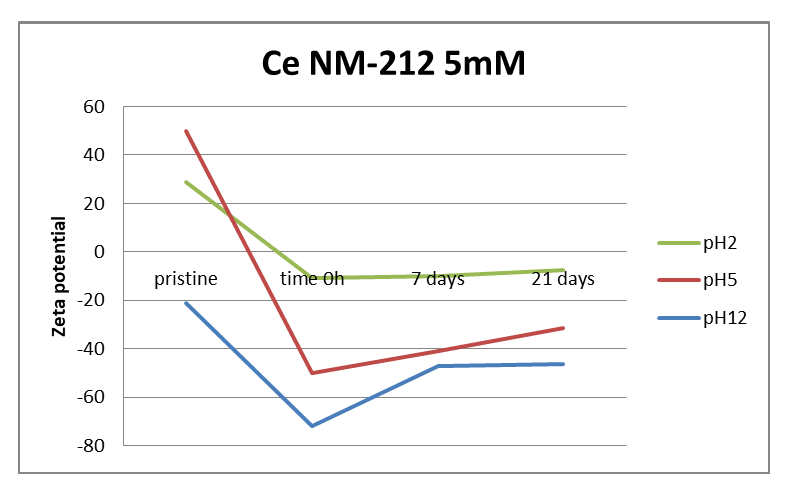

Supplement: S20 Fig — Different pH values and different exposure time points are shown. (TIFF) [file pone.0217483.s020.tiff]

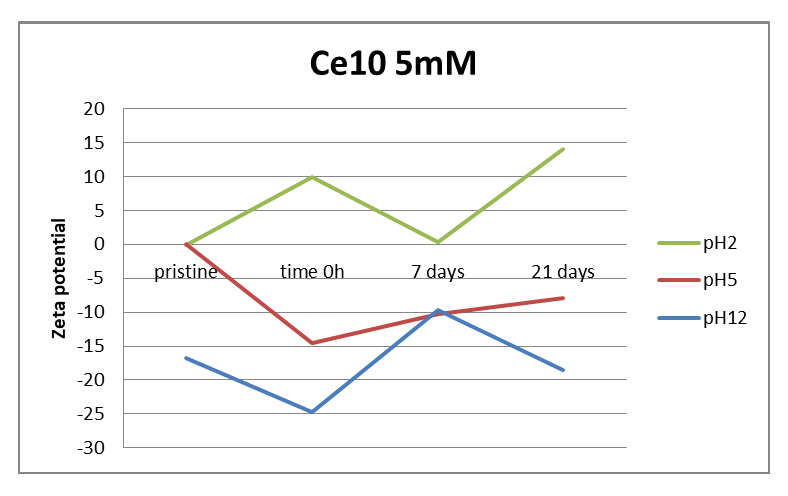

Supplement: S21 Fig — Different pH values and different exposure time points are shown. (TIFF) [file pone.0217483.s021.tiff]
